# Dietary *Lactobacillus fermentum* and *Lactobacillus paracasei* improve the intestinal health of broilers challenged with coccidia and *Clostridium perfringens*

**DOI:** 10.3389/fvets.2022.1025677

**Published:** 2022-12-15

**Authors:** Peng Li, Liyun Zheng, Ya Qi, Zhipeng Liu, Encun Du, Jintao Wei, Zhengfan Zhang, Shuangshuang Guo, Binying Ding

**Affiliations:** ^1^Hubei Key Laboratory of Animal Nutrition and Feed Science, Wuhan Polytechnic University, Wuhan, Hubei, China; ^2^Key Laboratory of Animal Embryo Engineering and Molecular Breeding of Hubei Province, Institute of Animal Science and Veterinary Medicine, Hubei Academy of Agricultural Sciences, Wuhan, China

**Keywords:** *Lactobacillus fermentum*, *Lactobacillus paracasei*, intestinal health, broiler, necrotic enteritis

## Abstract

Necrotic enteritis (NE) is a great threat to the intestinal health of broilers, resulting in decreased growth performance and significant economic losses. *Lactobacillus fermentum* (*LF*) and *Lactobacillus paracasei* (*LP*) exert beneficial effects on intestinal health. The aim of the present study was to investigate the effects of dietary *LF* and *LP* on the intestinal health and growth performance of broilers challenged with coccidia and *Clostridium perfringens* (CCP). The animal trial was carried out using 336 broilers (Ross 308) for 35 days with a completely randomized design. The broilers were divided into 4 groups based on treatment as follows: the control (CTR) group was fed the basal diet and without CCP challenge and the CCP group was fed the basal diet and with CCP challenge. The broilers in the CCP+*LF* and CCP+*LP* groups were challenged by CCP, and meanwhile, *LF* (1 × 10^9^ CFU/g) and *LP* (1 × 10^9^ CFU/g) were supplemented into the basal diets, respectively. The results showed that the growth performance and the intestinal morphology were negatively affected by the CCP challenge. In addition, the number of coccidia in the intestinal digesta and the relative abundance of *Escherichia coli* in the cecal digesta were increased. Besides, the mRNA level of *IgA* in the jejunum was downregulated, and the transcript level of *IL-8* was upregulated by the CCP challenge. Dietary *LF* and *LP* failed to improve the growth performance of broilers with the CCP challenge. However, they were beneficial for intestinal barrier function. In addition, dietary *LF* was able to alleviate the downregulation of *TGF-*β mRNA level in the spleen with CCP challenge and decreased the lesion scores compared with the CCP group. Furthermore, dietary *LP* alleviated the upregulation of the *IL-8* mRNA level in the jejunum with CCP challenge and reduced the number of coccidia in the ileal digesta. In conclusion, dietary *LF* and *LP* failed to mitigate the negative effects of CCP infection on growth performance; however, they were able to improve the intestinal health of broilers challenged with CCP by strengthening the intestinal barrier and alleviating inflammation.

## Introduction

Necrotic enteritis (NE) in broilers caused by *C. perfringens* is an enterotoxemic disease that shapes the impaired intestinal barrier, unstable intestinal flora, and weakened immunity of birds (1). In fact, NE results in significant economic losses. A study suggested that a loss of $0.05 per bird was caused by NE (2). Previously, antibiotics were used to treat NE in poultry production. However, antibiotics are banned in feed in many countries, and in recent years, the incidence of NE has been increasing. At present, nutrition-based interventions to address this problem have attracted the attention of many scholars. Probiotics have great potential to improve intestinal health, and dietary probiotics continue to be a good antibiotic substitute to alleviate NE in broilers. It was reported that *Lactobacillus fermentum* (*LF*) could produce short-chain fatty acids (SCFAs), which can inhibit the proliferation of *Escherichia coli* (3). Some studies also suggested that dietary *LF* was beneficial to the intestinal barrier and immunity of weaned piglets (4, 5). Another study demonstrated that *Lactobacillus paracasei* (*LP*) improved intestinal flora, which contributed to the growth performance and immunity of birds (6, 7).

Although some biological effects of *LF* and *LP* had been investigated, they were not fully characterized in poultry. The reported beneficial effects of *LF* and *LP* on intestinal health suggest their potential to alleviate NE. Hence, it was necessary to study the effects of dietary *LP* and *LF* supplementation in NE broilers. However, the lack of a reliable NE model might leave us stranded. A stable NE model in broilers should be of great importance. In our previous study, *C. perfringens* could be used in establishing the NE model (8, 9). However, due to differences in individual birds or dietary factors, the infection of *C. perfringens* was also not always successful to establish the NE model. A study suggested that the coinfection of coccidia and *C. perfringens* might make a more stable NE model (10). In the present study, coccidia and *C. perfringens* were used to establish a NE model, and dietary *LP* and *LF* were employed to prevent NE. This study aimed at investigating the effects of dietary *LF* and *LP* on the intestinal health and growth performance of broilers infected with NE.

## Materials and methods

### Animal and diet

**Figure 1:**
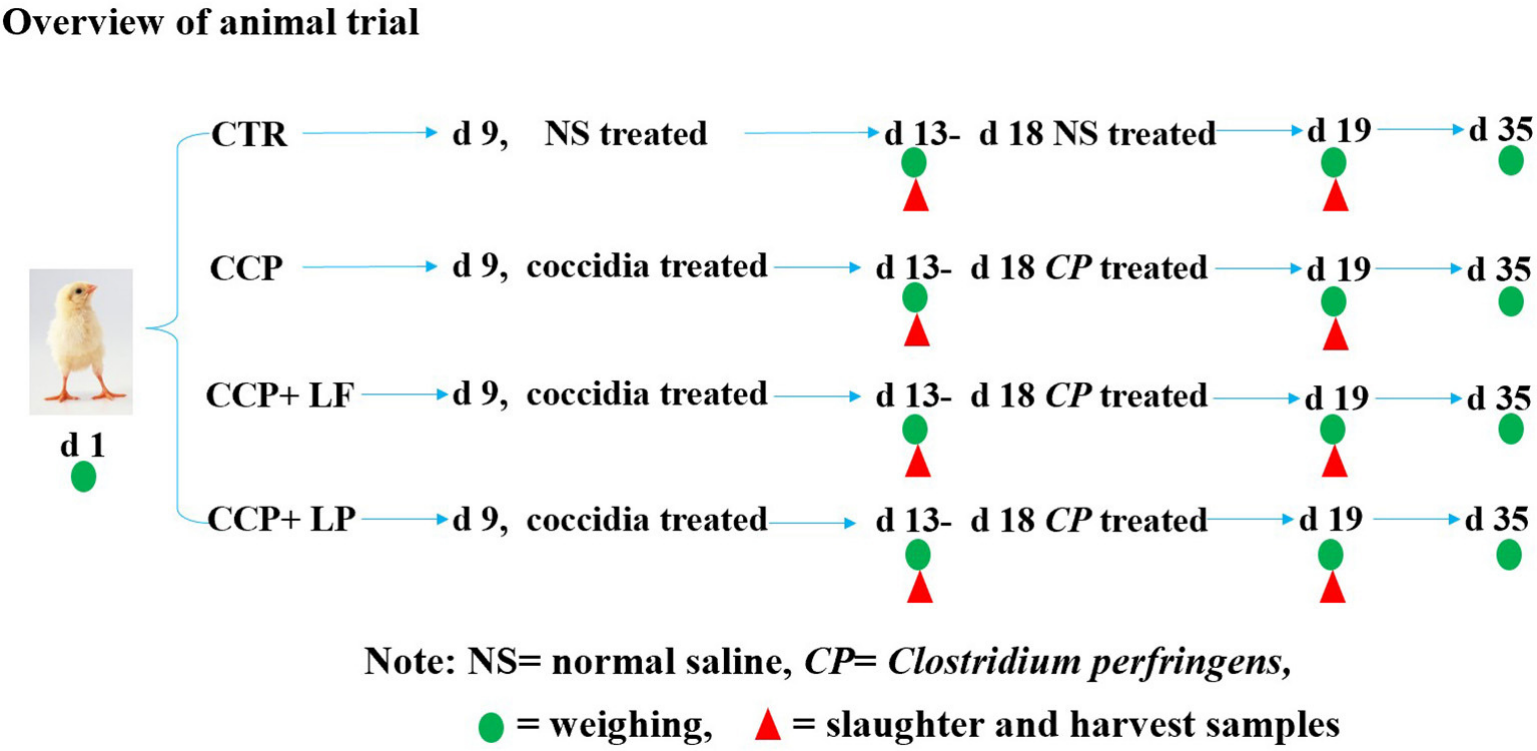
The schematic diagram of the overview of the trial. All broilers were weighed to investigate the growth performance on days 1, 13, 19, and 35. Some broilers were selected to be slaughtered, and samples were collected for laboratory analysis. Among them, green circles indicate that all chickens were weighed and recorded on that day, and red triangles indicate the broiler chickens that were selected for slaughter and sampling on that day.

The animal study was carried out at the Wuhan Polytechnic University (Hubei, China). A total of 336 1-day-old healthy Ross 308 broilers with uniform weight were randomly assigned to 4 treatments. There were 6 replicates in each group and 14 broilers (7 males and 7 females) in each replicate. The broilers in the control (CTR) group were fed the basal diet and without coccidia and were affected by the *C. perfringens* (CCP) challenge. The basal diet was formulated according to the recommendation of the NRC (1994). The formula and nutrient levels of the basal diet are shown in Table 1. The broilers in the CCP group were fed the basal diet and challenged by CCP. The *C. perfringens* (CVCC2030) was purchased from the China Veterinary Microbial Culture Collection and Management Center (Beijing, China), and the coccidia were purchased from Foshan Zhengdian Biotechnology Co., Ltd. (Guangdong, China). A quadrivalent anti-coccidiosis vaccine consisted of 5 × 10^4^ oocysts of *E. tenella* strain PTMZ, *E. necatrix* strain PNHZ, and *E. maxima* strain PMHY, as well as 1 × 10^5^ oocysts of *E. acervuline* strain PAHY. The recommended dose of the vaccine was 1,100 ± 110 sporulated oocysts per bird. In the present study, the number of coccidia oocysts inoculating each bird was 30 times the recommended dose. Birds in the CCP+*LF* and CCP+*LP* groups were also challenged with CCP, and the diets of the CCP+*LF* and CCP+*LP* groups were supplemented with 1 × 10^9^ CFU/g *LF* and 1 × 10^9^ CFU/g *LP*, respectively. The diet for each group was formulated one time a week. A 35-day trial was performed, and an overview of the trial is shown in Figure 1. On day 9 of the trial, the anti-coccidiosis vaccine was administered into the crop of broilers in the CCP, CCP+*LF*, and CCP+*LP* groups. The birds in the CTR group were treated with an equal volume of saline. From days 13 to 18, the broilers in the CCP, CCP+*LF*, and CCP+*LP* groups were challenged with *C. perfringens*. Specifically, 64 ml of *C. perfringens* broth (1 × 10^8^ CFU/mL) was well mixed into 1,200 g feed of each group, which was then equally distributed to each replicate. Importantly, these feeds should be consumed within 2 h, and the birds in the CTR group were fed a diet with an equal volume of sterile broth. On days 13 and 19, two birds in each replicate were selected to collect blood from the wing vein and slaughtered for sample collection. On days 1, 13, 19, and 35, all broilers and feed were weighed for the calculation of growth performance. All broilers were raised in wire cages with free access to water and feed. During the first 3 days of the trial, the room temperature was controlled at 35°C, and then, the temperature was decreased by 1°C per day until it was maintained at 25°C. A 24-h light regime was implemented throughout the animal trial.

**Table 1:** The feed ingredient composition and nutrient levels, air-dried basis.

**Ingredient, %**	**d 0- 21**	**d 21- 35**	**Nutrition level^c^**		**d 0- 21**	**d 21- 35**
Wheat	68.69	68.78	ME	mc/kg	2.929	2.996
soybean meal	20.32	23.18	Crude protein	%	21.606	19.739
Fish meal	5.00	0.00	Lysine	%	1.156	1.000
Soybean oil	2.50	4.20	Methionine	%	0.603	0.458
CaHPO4	1.20	1.60	Methionine+ Cystine	%	0.914	0.760
Stone powder	1.10	1.10	Calcium	%	1.131	1.061
NaCl	0.35	0.35	Available phosphorus	%	0.482	0.402
DL-Met	0.26	0.18	Threonine	%	0.747	0.658
Choline chloride, 50%	0.20	0.20				
Mineral premix^a^	0.20	0.20				
L-Lys HCl, 78%	0.15	0.18				
Vitamin premix^b^	0.03	0.03				
Total	100.00	100.00				

a Trace element premix (provided per kilogram of feed) the following substances: Cu, 8 mg; Zn, 75 mg; Fe, 80 mg; Mn, 100 mg; selenium, 0.15 mg; iodine, 0.35 mg.

b Vitamin premix (provided per kilogram of feed) the following substances: vitamin A, 12,500 IU; vitamin D3, 2,500 IU; vitamin E, 18.75 mg; vitamin K3, 2.65 mg; vitamin B1, 2 mg; vitamin B2, 6 mg; vitamin B12, 0.025 mg; biotin, 0.0325 mg; folic acid, 1.25 mg; nicotinic acid, 50 mg; pantothenic acid, 12 mg.

c Calculated values.

### The determination of growth performance and the immune organ index

The body weight of the broilers and the feed consumption were weighed on days 1, 13, 19, and 35. The average daily gain (ADG), average daily feed intake (ADFI), and the feed conversion ratio (FCR) from days 1–13, days 14–19, days 20–35, and days 1–35 were calculated. The indexes of the spleen, the bursa of Fabricius, and the thymus on days 13 and 19 were also analyzed. The formula for calculating the immune organ index is as follows: immune organ index = the weight of organ (g) /the body weight of broiler (kg).

### The assay of immune and biochemical parameters in the serum

The blood was collected from the underwing vein and centrifuged at 3,000 r/min for 15 min at 4°C, and the serum was separated from the supernatant. The kits purchased from Nanjing Jiancheng Biotechnology Co., Ltd. (Jiangsu, China) were used to determine the levels of lysozyme, inductible nitric oxide synthase (i-NOS), and lactate dehydrogenase (LDH) in the serum. An automatic biochemical analyzer (Unicel DXC800, Beckman Coulter, USA) was used to analyze the levels of glucose (GLU), calcium (Ca), and phosphorus (P) in the serum.

### The measurement of intestinal morphology and lesion score

Approximately 1 cm of mid-segments of the duodenum, jejunum, and ileum were collected and fixed in 4% paraformaldehyde. Then, these intestinal segments were embedded in wax blocks and sectioned at 4 μm, and the sections were stained with eosin-hematoxylin. Intestinal villus height (VH) and crypt depth (CD) were measured as described in the previous study (11). Briefly, 10 straight and intact intestinal villi were randomly selected in each sample, and then the VH and CD were measured by an image analysis system using the Olympus BX-41TF microscope. The vertical distance from the tip of the villus to the crypt opening was considered as the villus height, and the vertical distance from the crypt opening to the ending was the crypt depth. The average values were calculated from the ten measurements, and the ratio of VH to CD was also calculated. In addition, the duodenum, jejunum, and ileum were cut lengthwise, and the digesta were rinsed. Then, a 6-point scoring system (12) was employed to score the intestinal lesion. The specific rules for this 6-point scoring were as follows: an intestine without any abnormalities should be a zero. The intestine marked 1 should be described as having a thin wall and diffuse fibrin attached to the mucosal surface of the intestine. There were 1–5 necrotic or ulcerated spots on the intestinal mucosa or deposits of fibrin that could not be removed, which should be marked as 2 points. The more severe the bowel lesion, the more spots there were. For example, 3 points for a number of lesions between 6 and 15, and 4 points for more than 16 lesions. The presence of a 2–3 cm patch of necrotic plaque on the intestine should be marked as 5 points. Beyond that, once the necrotic plaque penetrated from the intestinal mucosa into the lamina propria and muscle layers of the intestine, it was considered as 6 points.

### Coccidia counts in digesta and feces

The digesta of the jejunum, ileum, and cecum as well as the feces were collected on days 13 and 19, and then stored in a refrigerator at −20°C. A total of 2 g of samples were mixed with 10 ml of saline, and then 50 ml of saline was continuously added with stirring until there was no obvious fecal mass. A 60-mesh nylon was used to filter the mixture, and then the filtrate was collected and pipetted into the two counting chambers of the *M. mcswelli* counting plate. The chambers were filled with filtrate, and air bubbles should be removed. The counting plate was placed for 3 min before being counted using a microscope. The formula for calculating the data was as follows: number of coccidial oocysts per gram of feces (OPG) = [(n1 + n2)] × 60 × dilution factor/(2 × 0.15) × 2. Among them, n1 and n2 were the numbers of the coccidial oocysts in each chamber, and the volume of the counting chamber was 0.15 ml. The dilution factor was the number of times the mixture was diluted. The total volume of the mixture was 60 ml, and the weight of the digesta or feces was 2 g.

### Gene expression

The jejunum and the spleen were collected and placed in an RNase-free centrifuge tube and then snap-frozen in liquid nitrogen before being transferred to a refrigerator at −80°C. Extraction of total RNA, preparation of cDNA, and PCR were performed as previously described (13). The TRIzol reagent (Takara, Dalian, China) was used to obtain the total RNA, and the purity was checked with the following criteria: an OD_260_/OD_280_ ratio of ~2.0 and a 28 S/18 S rRNA ratio of > 1.8. Then, the gDNA Eraser (Takara, Dalian, China) was used to prepare the cDNA. An Applied Biosystems 7500 Fast Real-Time PCR System (Foster City, CA) was used to perform the qPCR. The 2^−ΔΔCT^ method (14) was used to analyze the relative expression of each gene, and the β*-actin* was used as the reference gene. Primers of the genes in the present study are listed in Table 2.

**Table 2:** List of gene primer sequences^a^.

**Gene name**	**Prime sequence (5^′^-3^′^)**	**NCBI number**
*TNF-α*	F-GAGCGTTGACTTGGCTGTC	NM_204267
	R-AAGCAACAACCAGCTATGCAC	
*IFN-γ*	F-AGCTGACGGTGGACCTATTATT	Y07922
	R-GGCTTTGCGCTGGATTC	
*IL-1β*	F-ACTGGGCATCAAGGGCTA	NM_204524
	R-GGTAGAAGATGAAGCGGGTC	
*TGF-β4*	F-CGGGACGGATGAGAAGAAC	M31160
	R-CGGCCCACGTAGTAAATGAT	
*IL-8*	F-ATGAACGGCAAGCTTGGA R-TCCAAGCACACCTCTCTTC	AJ009800
*IgA*	F-GTCACCGTCACCTGGACACCA	S40610
	R-ACCGATGGTCTCCTTCACATC	
*pIgR*	F-GGATCTGGAAGCCAGCAAT	AY233381
	R-GAGCCAGAGCTTTGCTCAG	
*Mucin-2*	F-TTCATGATGCCTGCTCTTGTG	XM_421035
	R-CCTGAGCCTTGGTACATTCTTGT	
*Claudin-1*	F-CATACTCCTGGGTCTGGTTGGT	AY750897.1
	R-GACAGCCATCCGCATCTTCT	
*ZO-1*	F-CTTCAGGTGTTTCTCTTCCTCCTC	XM_413773
	R-CTGTGGTTTCATGGCTGGATC	
*Occludin*	F-ACGGCAGCACCTACCTCAA	D21837.1
	R-GGGCGAAGAAGCAGATGAG	
*β-actin*	F-GAGAAATTGTGCGTGACATCA	NM_205518.1
	R-CCTGAACCTCTCATTGCCA	

a The primers were designed and synthesized by Shanghai Sangon Bioengineering Co., Ltd. Shanghai, China. TNF-α, tumor necrosis factor α; IFN-γ, interferon γ; IL-1β and IL-8, interleukin 1β and interleukin 8; TGF-β4, transforming growth factor β4; IgA, immunoglobulin A; pIgR, polymeric immunoglobulin receptor; ZO-1, zonula occludens protein 1.

### Analysis of specific intestinal microflora

The cecal digesta was collected on day 19. QIAamp fast DNA stool mini kits (Qiagen, Hilden, Germany) were used to extract the genomic DNA of the cecal microflora. A NanoDrop 2000 spectrophotometer (Thermo Scientific, USA) was used to measure the concentration of DNA. The steps of qPCR and the calculation method of genes were the same as described above. The total bacteria (16s rRNA) were used as the reference gene, and the specific 16S rRNA genes were targeted for *E. coli, Lactobacillus*, and *C. perfringens*. The primers of these bacteria were as follows: total bacteria: F-ACTCCTACGGGAGGCAGCAGT and R-GTATTACCGCGGCTGCTGGCAC; *E. coli*: F-GTTAATACCTTTGCTCATTGA and R-ACCAGGGTATCTAATCCTGT; *Lactobacillus*: F-AGCAGTAGGGAATCTTCCA and R-CACCGCTACACATGGAG; and *C. perfringens*: F-AAAGATGGCATCATCATTCAAC and R-TACCGTCATTATCTTCCCCAAA.

### Data analysis

A one-way ANOVA program in SPSS 23.0 software (SPSS Inc., Chicago, IL) was used to analyze the data, and then, Tukey's multiple comparisons were used to investigate the differences between groups. The Kolmogorov-Smirnov test was used to analyze the data that did not comply with normal distribution, and a non-parameter test and pairwise comparisons were used to analyze the data. The Mann-Whitney U test was used to analyze lesion scores between treatments. The data of the lesion score was expressed in the median and interquartile range, and data other than the lesion score were presented as mean ± standard error. A *P*-value of < 0.05 was considered to be significantly different between groups, and the Graphpad prism 8.0 software was used for creating graphs.

## Results

### Growth performance and serum parameters

The FCR during days 1–13 and 14–19 was negatively affected by the CCP challenge, as well as the ADG and ADFI during days 14–19 (*P* < 0.05) (Table 3). The growth performance in other periods was not significantly affected by CCP treatment. In addition, dietary *LF* and *LP* failed to alleviate the negative effects of CCP on growth performance.

**Table 3:** The growth performance of broilers during the trial, *n* = 6.

**Item**	**CTR**	**CCP**	**CCP+*LF***	**CCP+*LP***	***P-value***
**d 1-13**					
ADFI, g	28.57 ± 0.65	27.97 ± 0.29	28.95 ± 0.61	28.60 ± 0.51	0.631
ADG, g	21.65 ± 0.47	20.71 ± 0.15	21.60 ± 0.51	21.14 ± 0.34	0.317
FCR	1.32 ± 0.01^b^	1.35 ± 0.01^a^	1.34 ± 0.01^a^^b^	1.35 ± 0.01^a^	0.002
**d 14-19**					
ADFI, g	60.03 ± 1.53^a^	54.68 ± 0.68^b^	56.03 ± 0.90^a^^b^	53.80 ± 0.95^b^	0.003
ADG, g	40.30 ± 1.07^a^	34.68 ± 0.41^b^	35.31 ± 0.65^b^	33.64 ± 0.59^b^	< 0.001
FCR	1.49 ± 0.01^b^	1.58 ± 0.01^a^	1.59 ± 0.02^a^	1.60 ± 0.01^a^	< 0.001
**d 20-35**					
ADFI, g	99.54 ± 2.11	100.46 ± 1.76	103.30 ± 2.17	100.87 ± 2.59	0.660
ADG, g	57.44 ± 1.30	58.90 ± 1.55	60.39 ± 1.89	58.76 ± 1.62	0.643
FCR	1.73 ± 0.03	1.71 ± 0.01	1.71 ± 0.02	1.72 ± 0.01	0.790
**d 1-35**					
ADFI, g	63.56 ± 1.23	62.82 ± 0.60	64.63 ± 0.74	63.08 ± 1.19	0.583
ADG, g	39.57 ± 0.71	38.88 ± 0.59	39.96 ± 0.63	38.80 ± 0.76	0.576
FCR	1.61 ± 0.02	1.62 ± 0.01	1.62 ± 0.01	1.63 ± 0.01	0.722

a,b Means in the same row without common superscripts differ significantly (P < 0.05). Data were presented as mean ± standard error. CTR, control group; CCP, the infection model group that was challenged with Clostridium perfringens and coccidia, CCP+ LF, the infection model group with Lactobacillus fermentum supplemented in the diet; and CCP+ LP, the infection model group with Lactobacillus paracasei supplemented in the diet.

The index of the thymus on day 13 tended to be reduced (*P* = 0.091), and the spleen index on day 19 tended to be increased by CCP challenge (*P* = 0.093) (Table 4). Dietary *LF* and *LP* were not able to improve immune organ indices. The content of serum glucose on day 13 was decreased (*P* < 0.05), and serum lysozyme on day 19 tended to be increased by CCP challenge (*P* = 0.059) (Table 5). Additionally, compared with the CCP group, the levels of serum i-NOS on day 13 were increased in the CCP+*LF* group (*P* < 0.05). The contents of serum calcium on days 13 and 19 were elevated in the broilers of the CCP+*LP* group (*P* < 0.001).

**Table 4:** The immune organ indices, g/kg, *n* = 12.

	**Item**	**CTR**	**CCP**	**CCP+*LF***	**CCP+*LP***	***P-*value**
d13	Spleen	0.71 ± 0.06	0.83 ± 0.05	0.83 ± 0.05	0.79 ± 0.06	0.438
	Bursa of fabricius	2.18 ± 0.08	2.43 ± 0.18	2.22 ± 0.15	2.31 ± 0.10	0.562
	Thymus	1.74 ± 0.11^a^	1.53 ± 0.10^a^^b^	1.32 ± 0.07^b^	1.38 ± 0.06^b^	0.007
d19	Spleen	0.72 ± 0.05^b^	0.89 ± 0.05^a^^b^	0.98 ± 0.06^a^	1.06 ± 0.10^a^	0.007
	Bursa of fabricius	2.40 ± 0.12	2.14 ± 0.15	2.29 ± 0.18	2.24 ± 0.16	0.701
	Thymus	1.60 ± 0.12	1.45 ± 0.08	1.40 ± 0.08	1.59 ± 0.11	0.371

a,b Means in the same row without common superscripts differ significantly (P < 0.05). Data were presented as mean ± standard error. CTR, control group; CCP, the infection model group that was challenged with Clostridium perfringens and coccidia; CCP+ LF, the infection model group with Lactobacillus fermentum supplemented in the diet; and CCP+ LP, the infection model group with Lactobacillus paracasei supplemented in the diet.

**Table 5:** The levels of serum immune and biochemical parameters, *n* = 12.

	**Item**	**CTR**	**CCP**	**CCP+*LF***	**CCP+*LP***	***P-*value**
d13	lysozyme, U/mL	12.42 ± 1.58	11.47 ± 2.17	12.04 ± 1.62	9.65 ± 2.18	0.766
	iNOS, U/mL	5.00 ± 0.51^b^	5.55 ± 0.70^b^	8.53 ± 0.87^a^	7.62 ± 0.69^a^^b^	0.003
	GLU, mg/dL	205.86 ± 2.88^a^	192.21 ± 2.90^b^	200.38 ± 3.57^a^^b^	198.52 ± 3.20^ab^	0.033
	Ca, mg/dL	10.59 ± 0.17^b^	10.81 ± 0.20^b^	11.26 ± 0.23^b^	12.25 ± 0.27^a^	<0.001
	P, mg/dL	7.15 ± 0.15	7.14 ± 0.16	7.34 ± 0.08	7.61 ± 0.24	0.173
	LDH, U/L	682.14 ± 30.87	638.57 ± 31.54	639.58 ± 23.42	663.82 ± 31.19	0.680
d19	lysozyme, U/mL	22.94 ± 1.21^b^	29.57 ± 2.90^a^^b^	25.97 ± 1.82^a^^b^	30.18 ± 0.97^a^	0.019
	iNOS, U/mL	9.81 ± 0.21	9.69 ± 0.25	10.47 ± 0.19	9.78 ± 0.28	0.067
	GLU, mg/dL	223.22 ± 6.18^a^	206.78 ± 4.67^a^^b^	197.02 ± 6.75^b^	200.59 ± 7.05^a^^b^	0.024
	Ca, mg/dL	10.58 ± 0.13^b^	10.67 ± 0.19^b^	11.31 ± 0.26^b^	12.25 ± 0.17^a^	<0.001
	P, mg/dL	6.80 ± 0.10	6.86 ± 0.25	6.40 ± 0.18	6.69 ± 0.19	0.314
	LDH, U/L	644.08 ± 37.42	746.15 ± 85.14	629.61 ± 33.96	713.13 ± 34.67	0.350

a,b Means in the same row without common superscripts differ significantly (P < 0.05). Data were presented as mean ± standard error. CTR, control group; CCP, the infection model group that was challenged with Clostridium perfringens and coccidia; CCP+ LF, the infection model group with Lactobacillus fermentum supplemented in the diet; and CCP+ LP, the infection model group with Lactobacillus paracasei supplemented in the diet.

### The measurement of the intestinal mucosa and cecal flora

**Figure 2:**
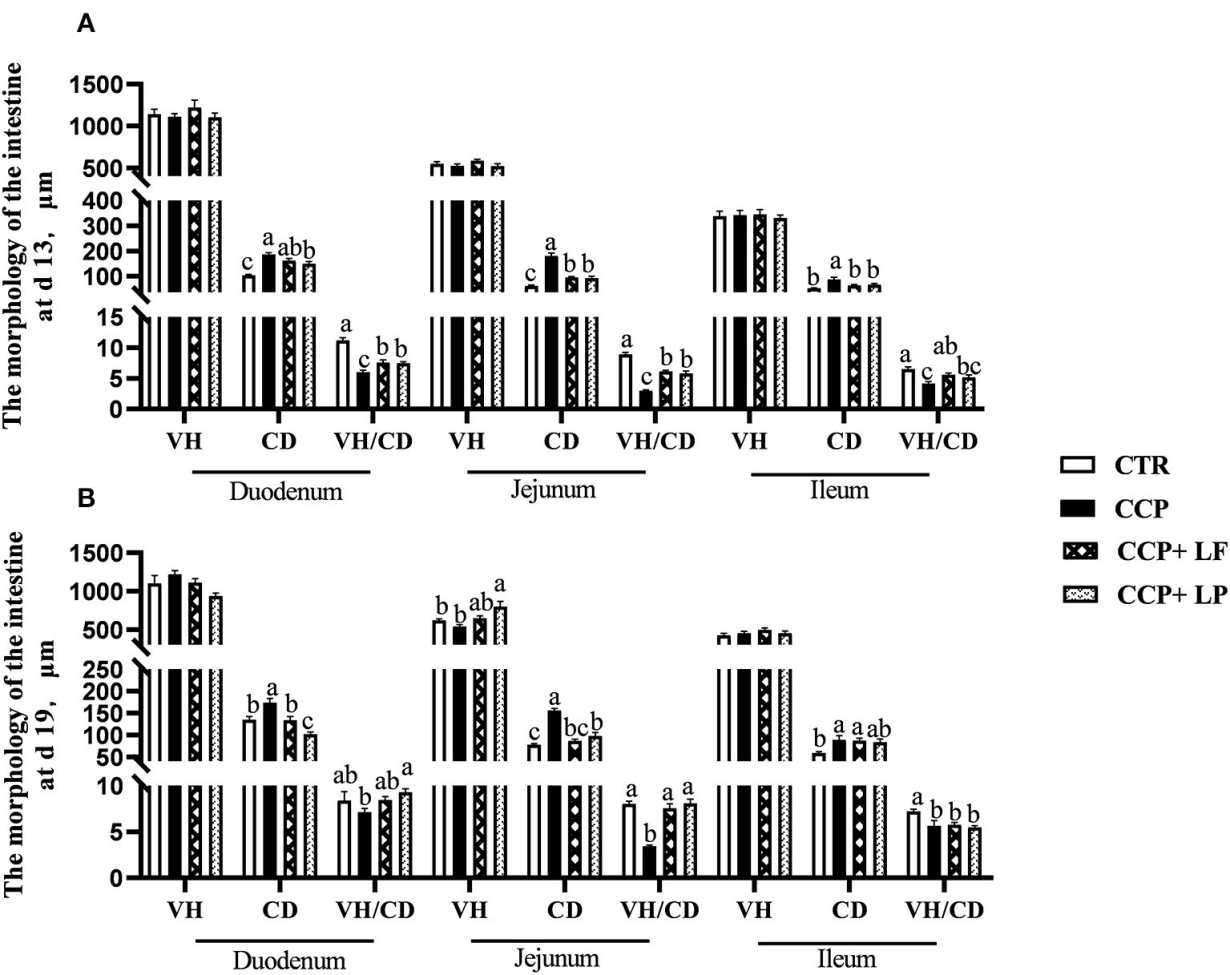
The intestinal villus height, crypt depth, and the ratio of villus height to crypt depth. **(A)** the data were collected on day 13, and **(B)** the data were collected on day 19. Among them, ^a,b^, and ^c^ means in the different pillars without common superscripts differ significantly (*P* < 0.05). VH, villus height; CD, crypt depth; VH/CD, the ratio of VH to CD. *n* = 12. Data were presented as mean ± standard error. CTR, the control group; CCP, the group co-infected with coccidia and *C. perfringens* (CCP), CCP+*LF* and CCP+*LP* denote the groups fed *Lactobacillus fermentum*- and *Lactobacillus paracasei*-supplemented diets with *CCP* challenge.

The crypt depths of the duodenum, the jejunum, and the ileum on days 13 and 19 were increased by CCP challenge (*P* < 0.05) (Figure 2). In addition, the ratios of VH to CD in the duodenum, the jejunum, and the ileum on day 13 and in the jejunum and the ileum on day 19 decreased. Dietary *LF* and *LP* elevated the ratios of VH to CD in the duodenum and the jejunum on day 13 and in the jejunum on day 19 of broilers with CCP challenge. Moreover, dietary *LF* increased the ratio of VH to CD in the ileum on day 13, and dietary *LP* improved it in the duodenum on day 19 with the CCP challenge.

**Figure 3:**
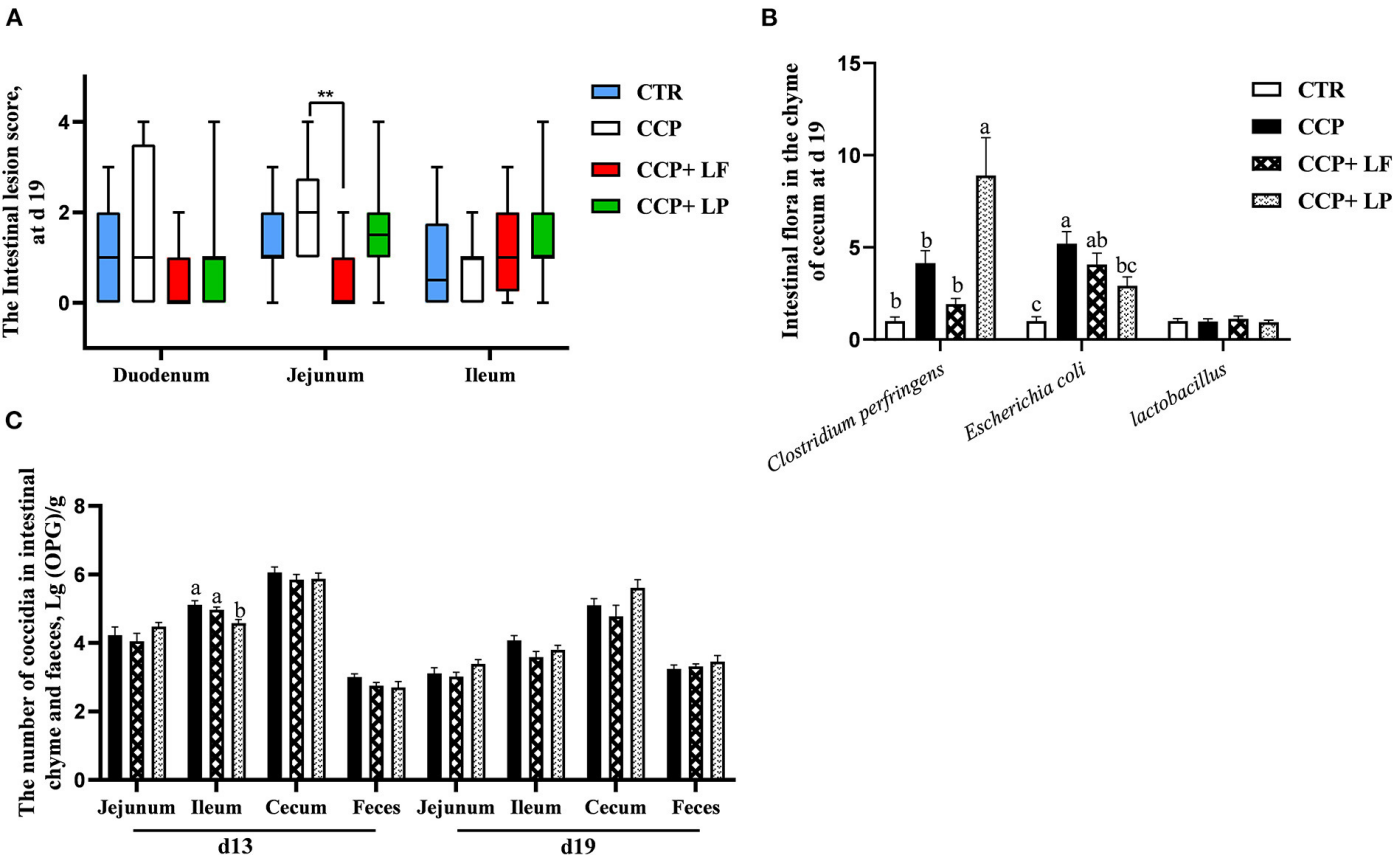
The intestinal lesion score, the number of cecal bacteria, and intestinal coccidia. The data of the intestinal lesion score is arranged in **(A)** (*n* = 12), the levels of some bacteria in the cecal chyme are shown in **(B)** (*n* = 10), and the numbers of coccidia in the chyme are arranged in **(C)** (*n* = 12 in the chyme of jejunum, ileum, and chyme. *n* = 6 in the feces). The data in **(A)** were expressed in the median and interquartile range, and data in **(B,C)** were presented as mean ± standard error. CTR, the control group; CCP, the group coinfected with coccidia and *C. perfringens* (CCP), CCP+*LF* and CCP+*LP* denote the groups fed *Lactobacillus fermentum*- and *Lactobacillus paracasei*-supplemented diets with *CCP* challenge. In **(A)**, ** = 0.001 < *P* < 0.01, among **(B,C)**, ^a,b^, and ^c^ indicate that the different pillars without common superscripts differ significantly (*P* < 0.05).

The CCP+*LF* group had a lower lesion score in the jejunum than that of the CCP group (*P* < 0.05) (Figure 3A). The relative abundance of *E. coli* in the cecal digesta was upregulated by CCP challenge (*P* < 0.05) (Figure 3B), while dietary *LP* decreased it. Although we did not observe a statistical difference in the relative abundance of *C. perfringens*, it was numerically raised by the CCP challenge. Unexpectedly, dietary *LP* upregulated the relative abundance of *C. perfringens* in the cecal digesta. Compared with the CCP group, the number of coccidial oocysts in the ileal digesta of the CCP+*LP* group on day 13 was decreased (*P* < 0.05) (Figure 3C). It was concluded that dietary *LF* and *LP* exerted beneficial effects in CCP-challenged birds.

### The transcription levels of some genes in the jejunum and spleen

**Figure 4:**
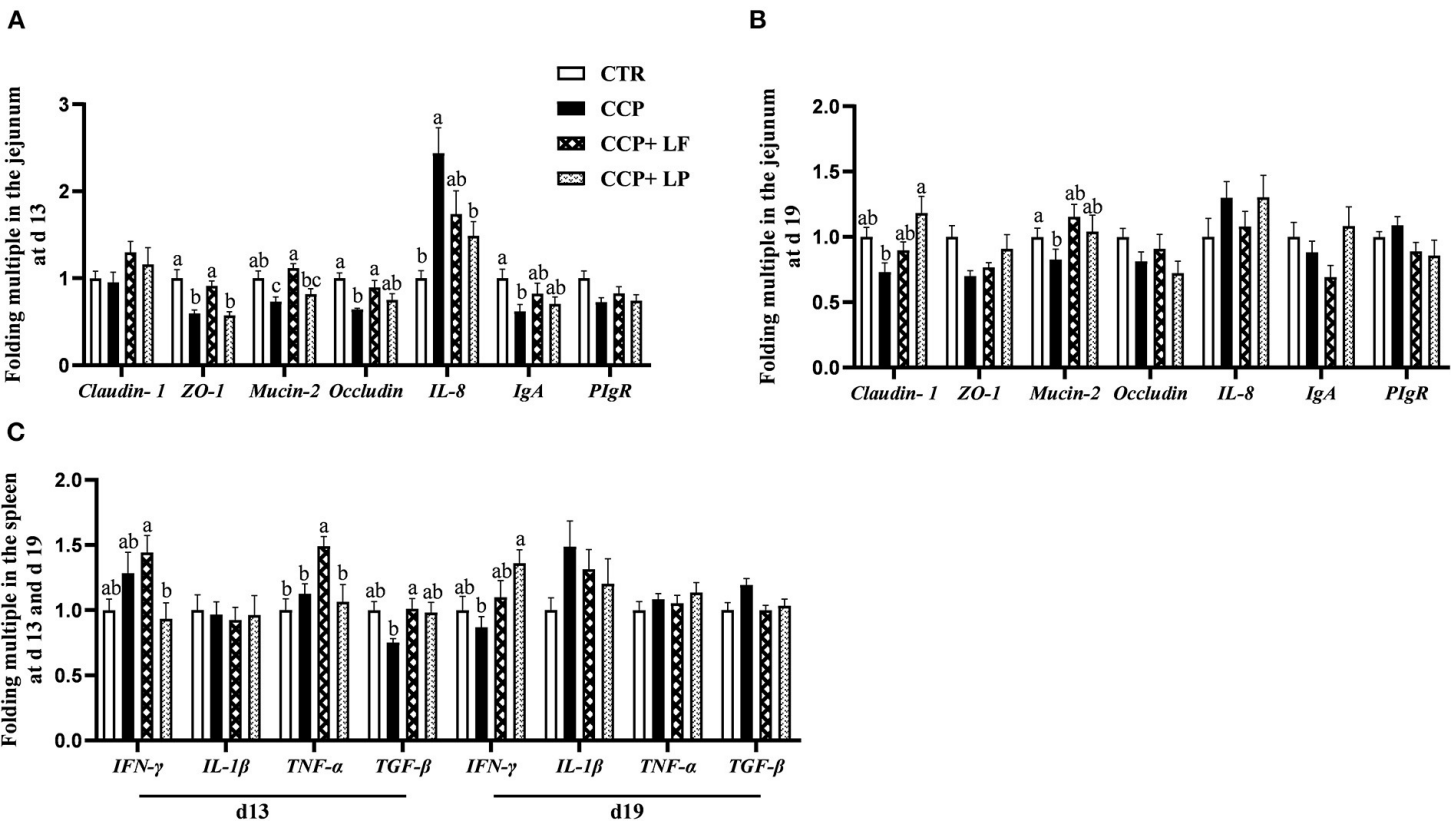
The mRNA transcript levels of some genes in the jejunum and spleen. **(A)** The data of the gene mRNA levels in the jejunum on day 13, **(B)** the data of the gene mRNA levels in the jejunum on day 19, and **(C)** the data of the gene mRNA levels in the spleen. Among them, ^a,b^, and ^c^ means in the different pillars without common superscripts differ significantly (*P* < 0.05). *n* = 12. Data were presented as mean ± standard error. CTR, the control group; CCP, the group coinfected with coccidia and *C. perfringens* (CCP), CCP+*LF* and CCP+*LP* denote the groups fed *Lactobacillus fermentum*- and *Lactobacillus paracasei*-supplemented diets with *CCP* challenge.

The mRNA levels of *ZO-1, Mucin-2*, and *Occludin* in the jejunum on day 13 were downregulated by CCP challenge, as well as the transcript level of *Mucin-2* in the jejunum on day 19 (*P* < 0.05) (Figures 4A,B). These results were logically consistent with the findings that CCP challenge weakened intestinal morphology. Dietary *LF* upregulated the mRNA levels of *ZO-1* and *Mucin-2* in the jejunum of broilers with CCP challenge on day 13 (*P* < 0.05). Dietary *LP* elevated the mRNA level of *Claudin-1* in the jejunum of broilers with CCP challenge on day 19 (*P* < 0.05). Besides, the results of immune-related genes showed that the mRNA level of *IL-8* was upregulated on day 13, and the transcript level *IgA* in the jejunum was downregulated by CCP challenge (*P* < 0.05), as well as the *TGF-*β in the spleen (*P* = 0.094) (Figure 4C). Compared with the CCP group, the mRNA level of *IL-8* in the jejunum on day 13 was downregulated, and the mRNA level of *IFN-*γ in the spleen on day 19 was upregulated with *LP* supplementation (*P* < 0.05). Additionally, dietary *LF* upregulated the transcript level of *TGF-*β in the spleen of the birds with CCP challenge on day 13 (*P* < 0.05).

## Discussion

A wheat-based diet was proven to help establish the NE model with coccidia and *C. perfringens* challenge (1). We presented an undated report based on this idea in this study. Liu et al. (15) suggested that the growth performance of broilers with CCP challenge was negatively affected, and it might be recorded only during CCP infection. In the present study, we observed consistent outcomes. Specifically, combined infection with coccidia and *C. perfringens* reduced the growth performance of broilers, which might only be observed during the infection period. There was no difference in growth performance between CCP-challenged and unchallenged broilers in the period after infection. A possible explanation was that the immune system of broilers was used to maintain immune homeostasis during the CCP challenge and that numerous nutrients needed to be ingested for immune responses. Since then, the compensatory growth mechanism (16) has been activated to eliminate the difference in growth performance. Some probiotics were found to be useful for the growth performance and immunity of broilers (17, 18), which opened a new window for relieving the NE in broilers. Unexpectedly, we observed that *LF* and *LP* were ineffective in the growth performance of CCP-infected broilers in this study. It was speculated that *LF* and *LP* might not be able to colonize in the intestine of broilers, as we found in the present study that dietary *LF* and *LP* failed to elevate the relative abundance of *Lactobacillus*. This transient probiotic might only be beneficial to the intestine, and this effect was not sufficient to improve the growth performance. To investigate our conjecture, we would conduct further studies on the intestines of broilers.

A healthy intestine should be equipped with a complete morphological structure and a stable microbial environment (19), and the tight junctions underlie the molecular basis of the intestinal barrier (20). Additionally, the mucins secreted by goblet cells of the intestine are regarded as the first guardian of intestinal health (21). In the present study, the mRNA levels of *Occludin, ZO-1*, and *Mucin-2* were downregulated by the CCP challenge. It was demonstrated that the CCP challenge weakened the intestinal barrier, which was consistent with previous reports (1). Besides, the ratio of villi height to crypt depth decreased with the CCP challenge. It was generally known that the longer the intestinal villi, the stronger the intestinal absorption function. Additionally, the intestinal crypts were rich in immature cells that were not capable of absorption, and the increase in crypt depth might be detrimental to the absorption function of the intestine. The ratio of intestinal VH to CD was considered a reliable indicator for assessing intestinal absorptive function (22). The negative effects of the CCP challenge on growth performance might be attributed to its disruption of the intestinal barrier. Interestingly, dietary *LF* and *LP* contributed to improving the intestinal morphology of broilers with CCP challenge in the present study. This was demonstrated in a number of studies that short-chain fatty acids produced by *Lactobacillus* in the intestine were beneficial for intestinal development and immunity (23, 24), which might be the evidence that dietary *LF* and *LP* improved the intestinal barrier function in this study. Although *LF* and *LP* did not improve the growth performance of CCP-challenged broilers, they strengthened the intestinal barrier. It showed that a combination of probiotics and other additives might improve the growth performance. This deserved an in-depth investigation.

The large quantity of microbes in the intestine is involved in shaping intestinal physiology (19), and a stable microflora is especially important. A study suggested that the stability of intestinal flora was perturbed by the CCP challenge and that some pathogenic bacteria might be active (1, 8). Pathogenic bacteria, such as *E. coli*, contain components of the cell wall like lipopolysaccharides that cause inflammation in the body by activating the toll-like receptor signaling pathways (25). In the present study, the relative abundance of *E. coli* in the cecal digesta was raised with CCP challenge, and *C. perfringens* was also numerically increased. Additionally, the number of coccidial oocysts was elevated. Dietary *LP* was able to alleviate the upregulation of *E. coli*. However, the relative abundance of *C. perfringens* was upregulated with *LP* supplementation. This was a somewhat surprising result since it was expected that dietary *LP* might inhibit the proliferation of *C. perfringens* in the intestine. A possible explanation was that, although dietary *LP* inhibited the proliferation of *E. coli*, in fact, it also disturbed the intestinal flora. The unstable flora structure provided an opportunity for the colonization of *C. perfringens* in the intestine. However, it did not aggravate the negative effects of the CCP challenge on broilers. Dietary *LP* decreased the number of coccidial oocysts with CCP challenge. It seemed that dietary *LF* and *LP* might have the potential to improve the intestinal environment. A report demonstrated that almost 80% of the immune response was mediated by gut microbes (26). Hence, it could be interesting to investigate the immunity in broilers.

It was accepted that calcium contributed to the performance and immunity of poultry (27, 28). In the present study, dietary *LP* elevated the level of calcium in the serum. Additionally, the level of serum i-NOS was also raised with *LF* and *LP* treatments. Based on those findings, it might be concluded that dietary *LF* and *LP* were beneficial for the immunity of broilers. A study suggested that CCP challenge caused inflammatory responses in broilers (1, 8), and IL-8 was considered a chemokine that was essential for angiogenesis and inflammation (29). Additionally, IL-8 has attracted considerable attention as an immunomodulator in inflammatory responses (29). In the present study, the mRNA level of *IL-8* in the jejunum was upregulated with CCP challenge, and dietary *LP* could alleviate its upregulation. In addition, the transcript level of *TGF-*β in the spleen was downregulated with CCP challenge, and dietary *LF* also reversed this result. Pro-inflammatory and anti-inflammatory cytokines were involved in maintaining immune homeostasis. TGF-β was a pivotal member of the anti-inflammatory factor family (30). The present findings demonstrated that the CCP challenge disrupted immune homeostasis and promoted inflammatory responses. This could be one of the reasons for the abnormal immune organ indices in broilers challenged by CCP.

## Conclusion

Combined infection with coccidia and *C. perfringens* impaired the intestinal barrier function by disrupting the intestinal morphology and tight junctions. In addition, coinfection led to inflammation in broilers. All these adverse factors negatively affect the growth performance of broilers. Dietary *LF* and *LP* improved the intestinal health of broilers challenged with CCP by strengthening the intestinal barrier and alleviating inflammation. Dietary supplementation of *LF* and *LP* has great potential to alleviate NE in broilers. Here, a detailed investigation of the mechanisms of the regulatory effects of dietary LF and LP on intestinal barrier function and immunity needs to be implemented.

## Data availability statement

The original contributions presented in the study are included in the article/supplementary material, further inquiries can be directed to the corresponding authors.

## Ethics statement

The animal study was reviewed and approved by Institutional Animal Care and Use Committee of Wuhan Polytechnic University.

## Author contributions

BD and SG designed the study. PL wrote the manuscript. PL, LZ, YQ, ZL, ED, JW, and ZZ helped to collect and analyze experimental results. BD, SG, and PL participated in the writing and revision of the manuscript. All authors contributed to the data interpretation and approved the final version of the manuscript.
